# Pathogenic differences of cynomolgus macaques after Taï Forest virus infection depend on the viral stock propagation

**DOI:** 10.1371/journal.ppat.1012290

**Published:** 2024-06-11

**Authors:** Paige Fletcher, Chad S. Clancy, Kyle L. O’Donnell, Brianna M. Doratt, Delphine C. Malherbe, Joseph F. Rhoderick, Friederike Feldmann, Patrick W. Hanley, Ayato Takada, Ilhem Messaoudi, Andrea Marzi

**Affiliations:** 1 Laboratory of Virology, Division of Intramural Research, National Institute of Allergy and Infectious Diseases, National Institutes of Health, Hamilton, Montana, United States of America; 2 Rocky Mountain Veterinary Branch, Division of Intramural Research, National Institute of Allergy and Infectious Diseases, National Institutes of Health, Hamilton, Montana, United States of America; 3 Department of Microbiology, Immunology, and Molecular Genetics, College of Medicine, University of Kentucky, Lexington, Kentucky, United States of America; 4 Division of Global Epidemiology, International Institute for Zoonosis Control, Hokkaido University, Sapporo, Japan; 5 International Collaboration Unit, International Institute for Zoonosis Control, Hokkaido University, Sapporo, Japan; 6 One Health Research Center, Hokkaido University, Sapporo, Japan; 7 Department of Disease Control, School of Veterinary Medicine, University of Zambia, Lusaka, Zambia; University of Texas Medical Branch / Galveston National Laboratory, UNITED STATES

## Abstract

Taï Forest virus (TAFV) is a negative-sense RNA virus in the *Filoviridae* family. TAFV has caused only a single human infection, but several disease outbreaks in chimpanzees have been linked to this virus. Limited research has been done on this human-pathogenic virus. We sought to establish an animal model to assess TAFV disease progression and pathogenicity at our facility. We had access to two different viral stock preparations from different institutions, both originating from the single human case. Type I interferon receptor knockout mice were inoculated with TAFV stock 1 or stock 2 by the intraperitoneal route. Inoculation resulted in 100% survival with no disease regardless of viral stock preparation or infectious dose. Next, cynomolgus macaques were inoculated with TAFV stock 1 or stock 2. Inoculation with TAFV stock 1 resulted in 100% survival and robust TAFV glycoprotein-specific IgG responses including neutralizing antibodies. In contrast, macaques infected with TAFV stock 2 developed disease and were euthanized 8–11 days after infection exhibiting viremia, thrombocytopenia, and increased inflammatory mediators identified by transcriptional analysis. Histopathologic analysis of tissue samples collected at necropsy confirmed classic filovirus disease in numerous organs. Genomic differences in both stock preparations were mapped to several viral genes which may have contributed to disease severity. Taken together, we demonstrate that infection with the two TAFV stocks resulted in no disease in mice and opposing disease phenotypes in cynomolgus macaques, highlighting the impact of viral stock propagation on pathogenicity in animal models.

## Author summary

The 2014–2016 West Africa Ebola virus epidemic was the largest in history affecting 10 countries globally, highlighting concerns for future filovirus outbreaks. In contrast, Taï Forest virus (TAFV), another filovirus, has caused a single human case of infection originating from a TAFV disease outbreak in chimpanzees in West Africa which demonstrates transmission to humans. However, limited research has been performed on this human pathogen. We wanted to fill this knowledge gap and characterize disease as more filovirus outbreaks occur in West Africa.

We had access to two TAFV stock preparations with sequence differences from separate institutions, both originating from the single human case. The impact of sequence differences on TAFV pathogenicity was assessed in cohorts of mice and macaques by infection with either TAFV stock. While both stocks caused no disease in mice, macaques displayed opposing disease phenotypes. Infection with TAFV stock 1 resulted in 100% survival with minimal disease; in contrast, macaques infected with TAFV stock 2 developed fatal disease within 11 days. Genomic differences between stock preparations were associated with viral entry and genome replication which could contribute to disease severity. This study highlights the importance of viral genomic differences and their impact on viral pathogenicity.

## Introduction

The *Filoviridae* family consists of single-stranded, negative-sense RNA viruses which can cause severe hemorrhagic disease in humans and nonhuman primates (NHPs) [1]. There are currently three orthoebolaviruses that have caused recorded disease outbreaks in humans: Ebola virus (EBOV), Sudan virus (SUDV), and Bundibugyo virus (BDBV). Taï Forest virus (TAFV) is a lesser-known filovirus that has also caused human disease; however, there has only been a single non-lethal case to date [2].

TAFV emerged in the Parc National de Taï of Côte d’Ivoire, hence the former identification as species *Côte d’Ivoire ebolavirus* [3]. It was first discovered in 1994 following an outbreak of hemorragic disease in chimpanzees in West Africa [4] and a non-lethal human infection. The single human case was a 34-year-old ethologist who was exposed to TAFV during a wild chimpanzee necropsy. The individual presented with classic filovirus disease, but fully recovered six weeks later with intensive supportive care. Limited research has been performed with this human-pathogenic virus, but with the recent emergence of EBOV in West Africa we aimed to address existing knowledge gaps to inform outbreak response preparedness as it is the only previously detected filovirus in this region.

We had access to two different TAFV stock preparations with variances in their genome sequences, referred to here as TAFV stock 1 and TAFV stock 2, which were derived from the same human case. Due to the locations of the genomic differences between the TAFV stocks, we aimed to assess TAFV pathogenicity and establish a disease model for countermeasure efficacy studies. Since type I interferon receptor knockout (IFNAR^-/-^) mice are susceptible to wildtype filovirus infection [5], they were a rational first choice to assess TAFV stock-specific phenotypic differences. However, our results showed that TAFV caused no disease in IFNAR^-/-^ mice independent of the viral stock used for inoculation. A previous study demonstrated that cynomolgus macaques infected intramuscularly (i.m.) with TAFV resulted in classical filovirus disease with 3 out of the 5 macaques succumbing to disease [6]. Therefore, cynomologus macaques were used next to assess phenotypic differences between TAFV stock 1 and stock 2 for a potential disease model. Our results demonstrated that the two TAFV stocks caused opposing disease phenotypes in these NHPs with stock 1 causing minimal disease and stock 2 causing lethal disease.

## Results

### Genomic variations between TAFV stock 1 and stock 2

The wildtype TAFV isolate was derived from a single human-infected case from 1994. We had access to two TAFV stocks from different institutions which were both derived from this same case; referred herein as TAFV stock 1 and stock 2. TAFV stock 1 was received with mycoplasma contamination and underwent a cleanup process, but TAFV stock 2 was mycoplasma-free. Sequencing results of the two stocks identified genomic variations, with stock 1 having greater homology to NCBI reference NC_014372 while stock 2 was similar to NCBI reference KU182910 (S1 Table). There were 4 non-coding variations. However, there were a total of 7 variations spread across the coding regions of 3 TAFV viral proteins: glycoprotein (GP), viral protein 30 (VP30), and the RNA-dependent RNA polymerase “large” protein (L) (S1 Table). The GP contained 4 variations which could impact viral entry into the host and viral editing. Both VP30 and L contained 2 variations each which could be linked to viral replication. Due to the locations of these genomic variations between the TAFV stocks, we aimed to assess differences *in vitro* and also potential pathogenicity differences between the two TAFV stocks *in vivo*. Differences regarding replication properties between the two TAFV stocks *in vitro* were assessed by performing growth kinetics in Vero E6 cells. TAFV stock 1 grew significantly faster for the first few days compared to TAFV stock 2 indicating that TAFV stock 1 may carry mutations favoring *in vitro* growth. However, both endpoint titers reached similar levels by 6 days post-infection (dpi; S1 Fig).

### No disease after TAFV inoculation in IFNAR^-/-^ mice

**Fig 1:**
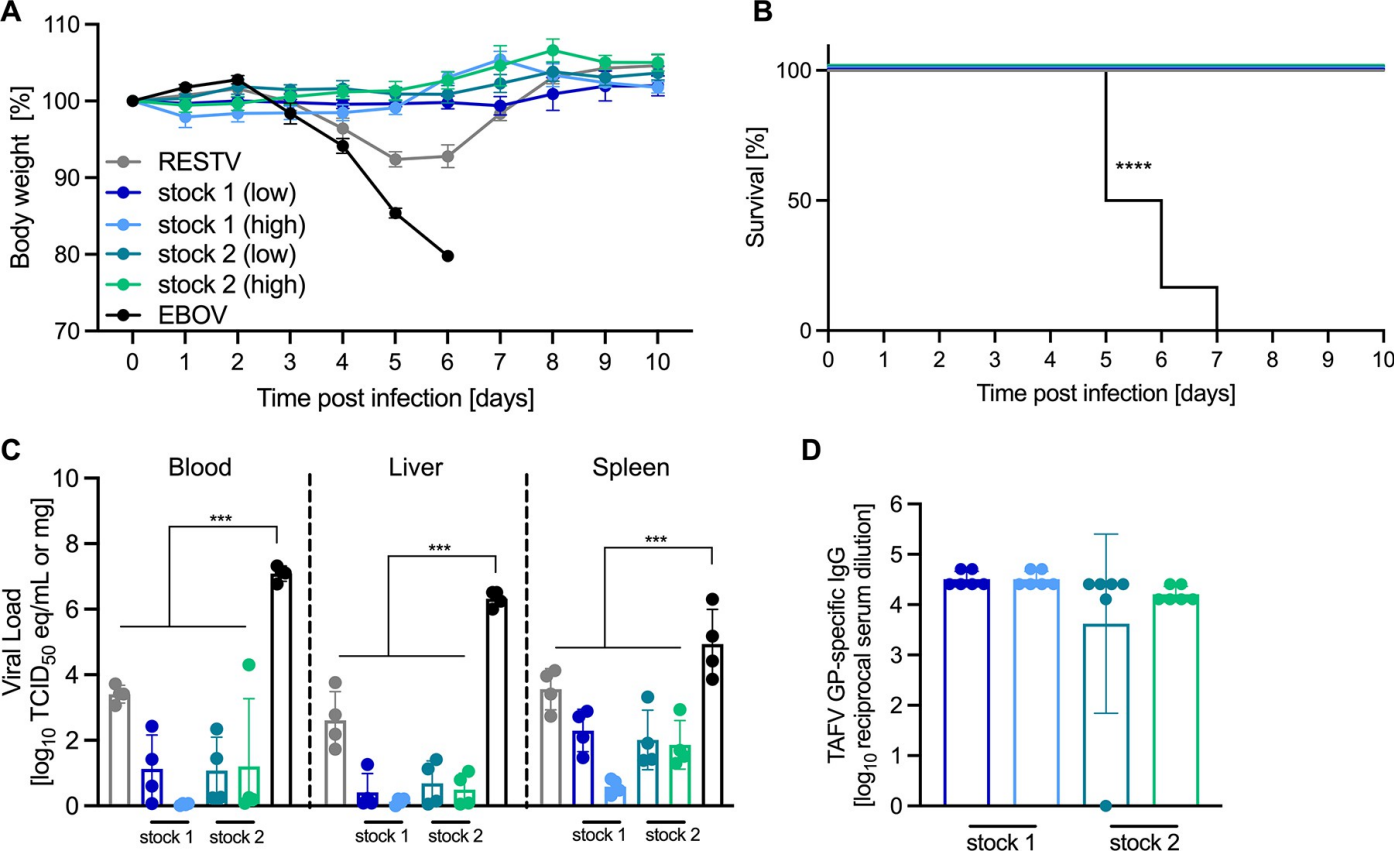
Intraperitoneal infection of TAFV in mice. IFNAR^-/-^ mice were infected i.p. with a low-dose (100 TCID_50_) of TAFV stock 1, 2, or EBOV or with a high-dose (100,000 TCID_50_) of TAFV stock 1, 2 or RESTV. At 6 dpi, 4 mice in each group were euthanized for viral load analysis. (A) Mouse body weight changes and (B) survival over time. (C) Viral RNA in blood, liver, or spleen. (D) TAFV GP-specific IgG in serum at study end (28 dpi). Mean and standard error of the mean are displayed in (A). Geometric mean and geometric standard deviation are displayed in (C, D). Statistical significance calculated by Mantel-Cox test or 2-way ANOVA with Tukey’s multiple comparisons is indicated as ***p<0.001 and ****p<0.0001.

With the intent to observe a disease phenotype in a rodent model for TAFV infection, we inoculated IFNAR^-/-^ mice with either a low (100 TCID_50_) or high (100,000 TCID_50_) dose of TAFV stock 1 or stock 2 via the i.p. route as this route of inoculation is commonly used for filovirus rodent models [7]. The inoculated mice remained healthy, gained weight regardless of TAFV stock and dose (Fig 1A), and survived (Fig 1B). Viral RNA levels were similar in mice inoculated with both TAFV stocks but lower than those in samples from RESTV- and EBOV-infected mice (Fig 1C) with the EBOV-infected mice succumbing to disease. All of the TAFV stock 1 mice and 11 out of 12 TAFV stock 2 mice seroconverted with TAFV GP-specific IgG present in serum at the study end (Fig 1D). Our results demonstrate that IFNAR^-/-^ mice are not an appropriate animal disease model for future studies.

### Differences in clinical disease after infection with TAFV stocks in NHPs

**Fig 2:**
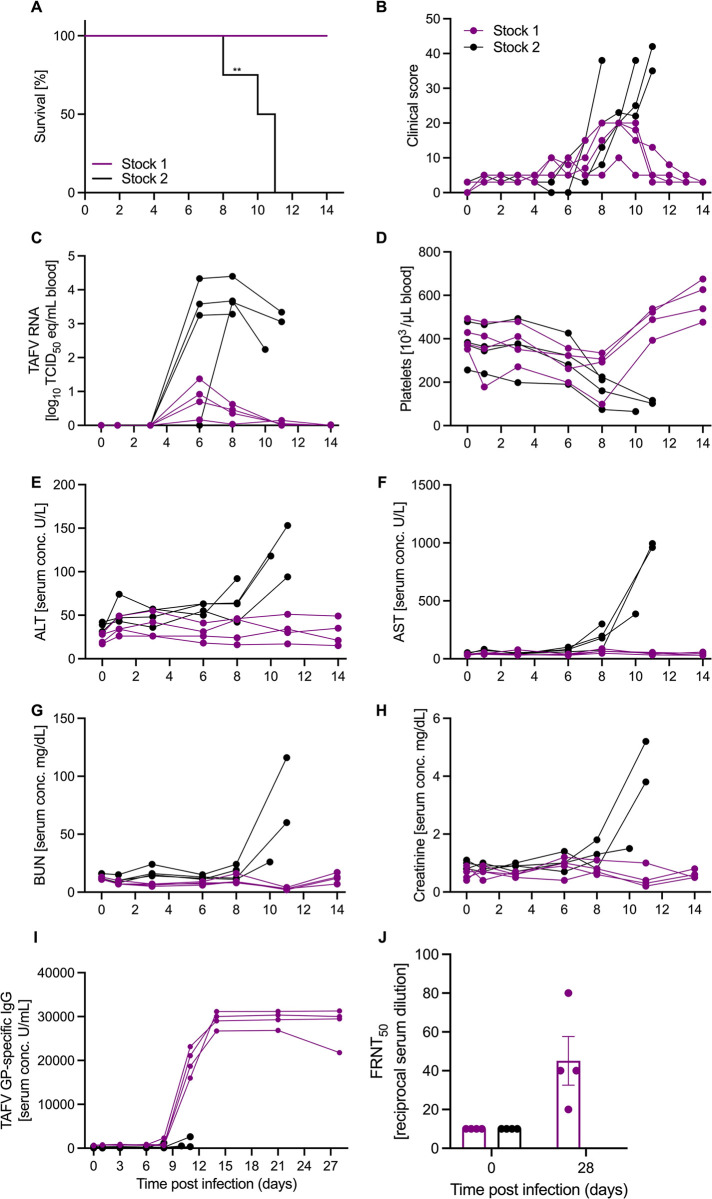
Clinical and serological findings in NHPs after TAFV infection. Cynomolgus macaques (n = 8) were intramuscularly infected with 1x10^3^ PFU of either TAFV stock 1 or stock 2. (A) Survival curve, (B) clinical score, (C) TAFV RNA in the blood, (D) platelet counts, and (E-F) liver and (G-H) kidney enzyme levels. (I) TAFV GP-specific IgG levels in serum. (J) Serum neutralization presented as 50% reduction of GFP-positive cells (FRNT_50_) at the time of infection (day 0) and euthanasia (day 28; study end). ALT, alanine aminotransferase; AST, aspartate aminotransferase; BUN, blood urea nitrogen; CREA, creatinine. Statistical significance calculated by Mantel-Cox test is indicated as **p<0.01.

Previous data demonstrated that cynomolgus macaques are susceptible to TAFV infection [6], therefore, we used this model next to assess potential differences between TAFV stock pathogenicity and disease. Groups of 4 NHPs were infected i.m. with 10,000 TCID_50_ of either TAFV stock 1 or stock 2 (stock 2 NHP data partially published previously in [8]). All stock 1 NHPs survived the length of the study with developing minimal signs of disease (Fig 2A–2B). Minimal levels of TAFV RNA were detected in the blood of stock 1 NHPs between 6 and 8 dpi, however, blood platelet counts remained stable (Fig 2C–2D). We did not detect any signs of TAFV shedding in the swab samples. There were no differences in levels of liver enzymes, renal biomarkers, or serum cytokines compared to baseline (Figs 2E–2H and S1). All NHPs infected with TAFV stock 1 developed a TAFV GP-specific IgG response measured in serum which peaked around 14 dpi and remained constant through the duration of the study (Fig 2I). The neutralizing activity in serum increased as expected from 0 to 28 dpi (Fig 2J).

In contrast, the 4 NHPs infected with stock 2 met the endpoint criteria between 8 and 11 dpi and were euthanized. These NHPs presented with characteristic filovirus disease including viremia (Fig 2C), thrombocytopenia (Fig 2D), elevated liver and kidney enzyme levels (Fig 2E–2H), and a dysregulated chemokine and cytokine response indicative of a cytokine storm (S2 Fig). Finally, we could not detect a TAFV GP-specific humoral immune response for TAFV stock 2 NHPs throughout the study (Fig 2I and 2J).

### Transcriptomic changes in NHPs after TAFV infection reflects disease severity

**Fig 3:**
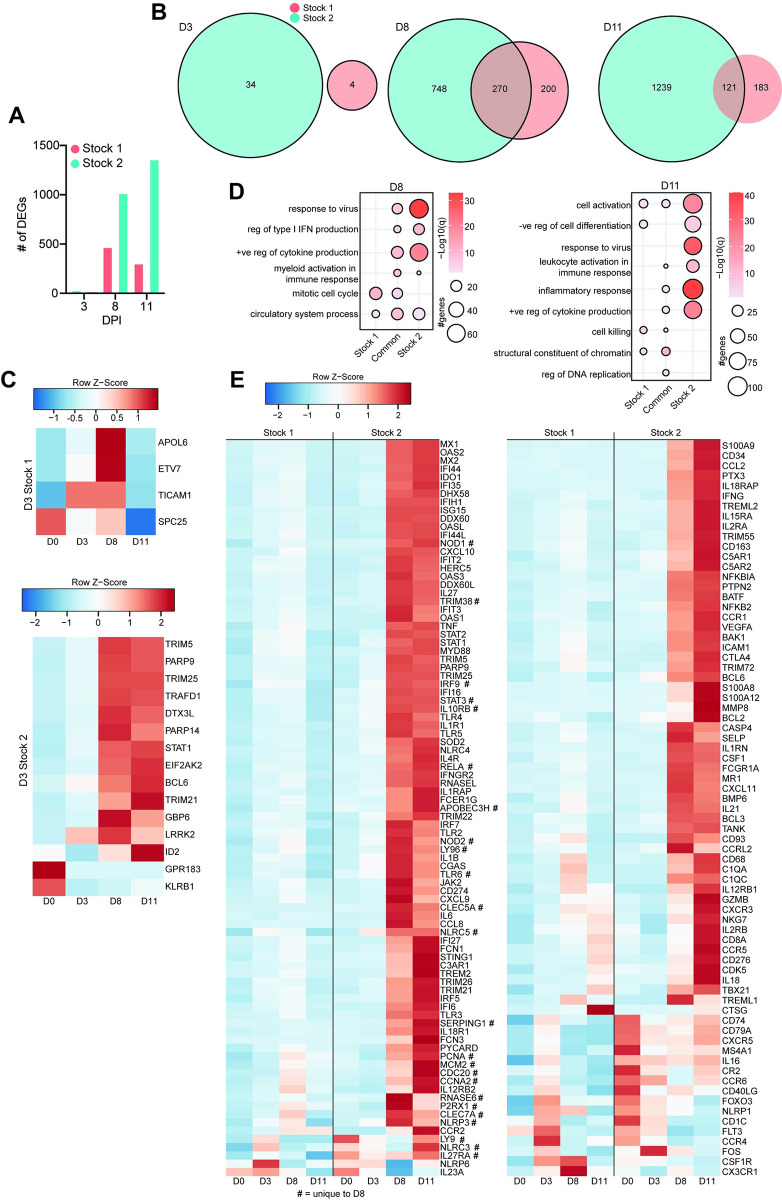
Transcriptional response of NHPs to TAFV infection. (A) Bar graph of the number of DEG identified at 3, 8, and 11 dpi relative to 0 dpi for TAFV stocks 1 and 2. (B) Venn diagram of DEG for 3 (top), 8 (middle), and 11 (bottom) dpi relative to 0 dpi. (C) Heatmap of average TPM values for stock 1 (top) and stock 2 (bottom) at 3 dpi. (D) Bubbleplot representing GO terms for 8 (left) and 11 (right) dpi relative to 0 dpi. Color indicated -log10(q) and size indicated the number of genes within the GO term. (E) Heatmap of average TPM values for 8 and 11 dpi (left) and unique to 11 dpi (right) from panel D. Color is based on scaled and centered TPM values in (C, E). #, unique to TAFV stock 2 at 8 dpi.

**Fig 4:**
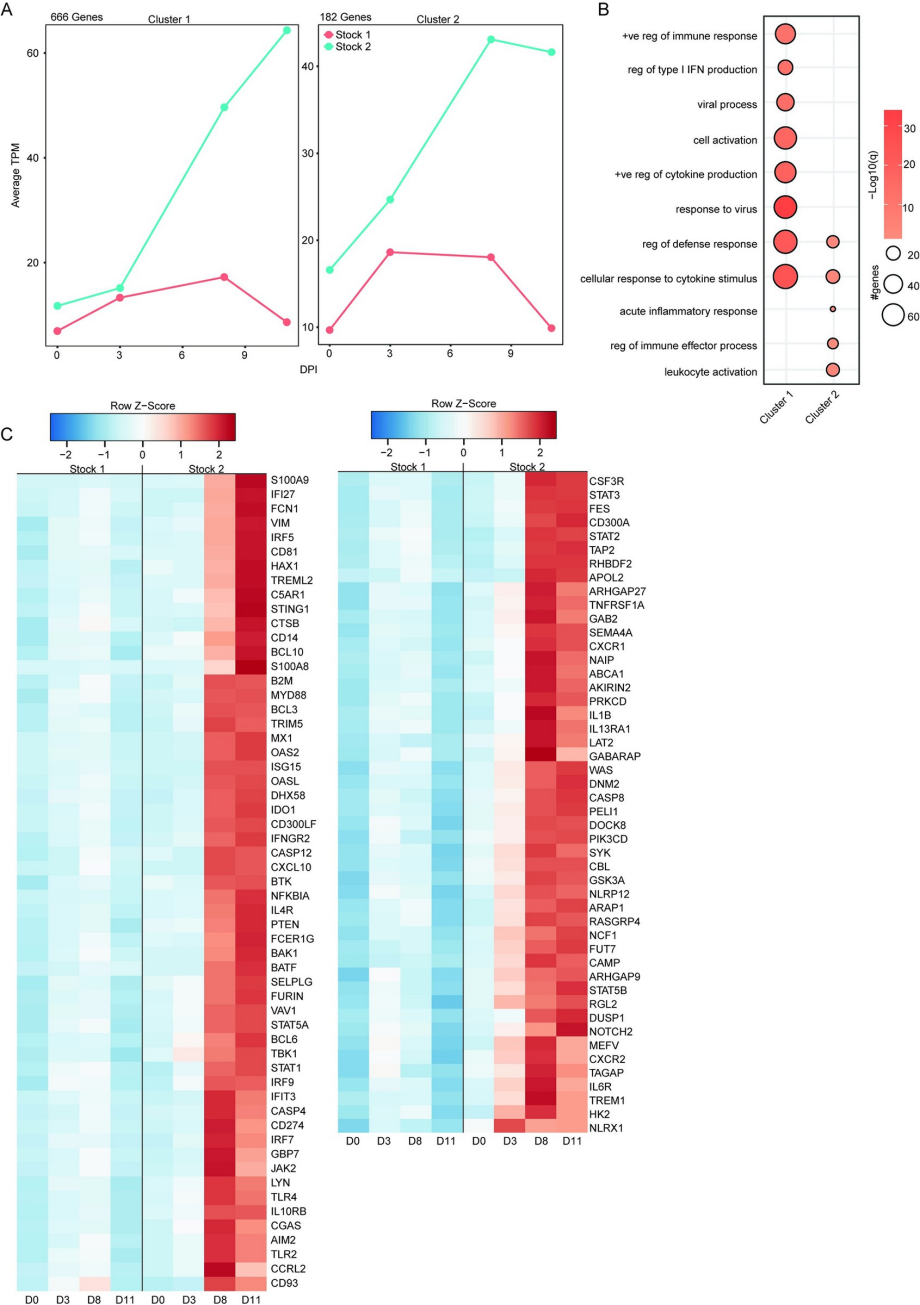
MaSigPro analysis of transcriptional changes between TAFV stocks. (A) Average TPM of the gene expression clusters identified by MaSigPro using two-way forward regression analysis. (B) Bubbleplot representing GO terms for genes from Cluster 1 and 2 from panel A. Color indicated -log10(q) and size indicated the number of genes within the GO term. (C) Heatmap of average TPM values mapping to GO terms in Panel B for Cluster 1 (left) and Cluster 2 (right). Color is based on scaled and centered TPM values.

Differences between TAFV stock 1 and stock 2 NHPs were assessed by transcriptomic analysis of RNA from whole blood samples collected during the acute disease phase (Fig 3). The number of differentially expressed genes (DEGs) was higher in stock 2 NHPs compared to stock 1 NHPs (Fig 3A). There were no common DEGs between the two TAFV stocks at 3 dpi (Fig 3B and 3C); however, the total number of common DEGs increased at 8 and 11 dpi. Although some DEGs shared between the NHPs infected with either TAFV stock mapped to similar gene ontology (GO) terms for 8 and 11 dpi, the number of DEGs and the enrichment was lower in the stock 1 NHPs (Fig 3D). Moreover, DEGs uniquely upregulated by stock 1 at 8 and 11 dpi mapped to GO terms associated with “mitotic cell cycle” (*MCM2*, *PCNA*, *CDC20*) and “cell killing” (*GZMB*, *CTSG*, *IL18*) (Fig 3D and 3E). NHPs infected with stock 2 had a greater increase of DEGs mapped to inflammatory processes at 8 and 11 dpi, such as “response to virus” (*MX1*, *STING1*), “regulation of type I interferon production” (*IRF7*, *IFNGR2*, *IRF5*), and “positive regulation of cytokine production” (*STAT2*, *TLR3*, *CXCL9*) (Fig 3D and 3E). The DEGs that were downregulated in the stock 2 NHPs at 8 and 11 dpi included host defense response (*NLRP6*, *IL23A*), innate immune response (*CD1C*, *CX3CR1*), and chemokine response (*CCR6*, *CCR4*) (Fig 3E). To better distinguish the responses between the two groups, we used MaSigPro software to identify longitudinal gene expression signatures (Fig 4). This analysis identified 2 clusters with temporal DEG changes vastly different between the two NHP groups as indicated by the steady increase of DEGs in stock 2 NHPs over time while stock 1 NHP DEGs started to decrease around 11 dpi (Fig 4A). Regardless of the cluster, the most pronounced increase in DEGs was observed for the stock 2 NHP group on 8 and 11 dpi. DEGs associated with cluster 1 mapped to GO terms “cell activation” (*CD81*, *CD274*, *CXCL10*, *FCER1G*), “regulation of type I interferon production” (*MYD88*, *TLR2*, *IRF5*, *OAS2*), and “response to virus” (*IFNGR2*, *STING1*, *AIM2*, *GBP7*, *TBK1*) (Fig 4B and 4C). DEGs within cluster 2 were associated with “leukocyte activation” (*CD300A*, *IL13RA1*, *NOTCH2*, *IL6R*) and “regulation of immune effector processes” (*STAT3*, *STAT5B*, *CXCR1*, *NCF1*) (Fig 4B and 4C).

To understand the protective response from NHPs infected with TAFV stock 1, we performed longitudinal GO analysis within these NHPs only (S3 Fig). GO terms at 8 dpi were the highest (S3A Fig) and associated with “innate immune response” (*SERPING1*, *IFI27*, *IRF7*, *STAT1*), “myeloid leukocyte activation” (*MYD88*, *TLR3*, *TNF*), and “DNA damage response” (*E2F1*, *CCND1*, *PCNA*) (S3B and S3C Fig). These transcriptional changes appeared to be associated with several transcription factors related to innate immune responses and cytokine stimuli (*IRF3*, *MAFK*, *FOS*) (S3D Fig). Lastly, we performed Short Time-series Expression Miner (STEM) analysis to gain a longitudinal insight into the inflammatory response for NHPs infected with TAFV stock 2 (S4 Fig). Transcriptional changes identified 4 clusters of DEGs in these NHPs with expression levels increasing on 8 and 11 dpi (S4A Fig). Genes mapped to GO terms “cytoplasmic translation” (*RPS8*, *RPS12*, *RPL13*, *RPL8*), “negative regulation of viral process” (*IFITM1*, *MX2*, *OAS1*, *OAS3*, *OASL*), “transcription factor binding” (*JAK3*, *NFKB1A*, *IKBKE*, *IKZF3*), and “regulation of inflammatory response” (*TREM1*, *MMP9*) (S4B and S4C).

### Histopathologic analysis of TAFV stock 2 NHPs revealed classic filovirus disease

**Fig 5:**
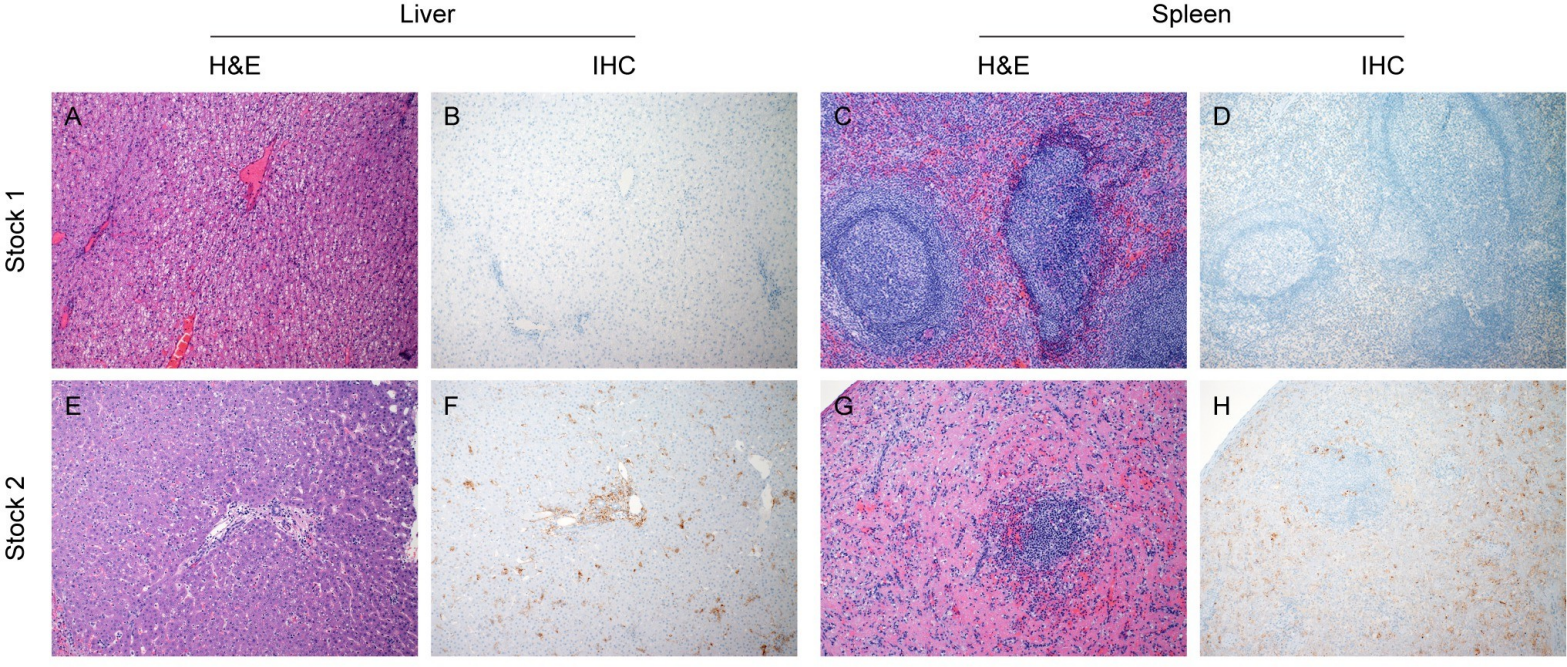
Histopathology and immunohistochemistry (IHC) of liver and spleen samples. Tissue samples were collected at the time of euthanasia and stained with hematoxylin & eosin (H&E) or for TAFV antigen immunoreactivity. (A, B) TAFV stock 1 liver; (C,D) TAFV stock 1 spleen; (E,F) TAFV stock 2 liver; (G,H) TAFV stock 2 spleen. All images are 100x.

The liver and spleen are primary target tissues of filovirus infection and greatly impacted during acute disease; therefore, histopathologic analysis was performed. It is important to keep in mind that TAFV stock 1 NHPs were euthanized at the study end (28 dpi) while TAFV stock 2 NHPs met endpoint criteria between 8 and 11 dpi. No evidence of TAFV infection was observed in histopathologic evaluation or TAFV-specific immunoreactivity of the liver or spleen in any of the NHPs infected with TAFV stock 1 (Fig 5A–5D). In contrast, histopathologic lesions consistent with classically described filovirus disease were observed in 4/4 NHPs infected with TAFV stock 2. Lesions included widely disseminated, random, small foci of hepatocellular necrosis and histiocytic infiltration throughout the liver associated with immunoreactivity (Fig 5E and 5F) and lymphoid follicular necrosis in the spleen similarly associated with immunoreactivity (Fig 5G and 5H). Since little is known about TAFV pathogenesis in this model, we performed a thorough histopathologic analysis of tissue samples collected throughout the NHP. No significant histopathologic changes attributable to TAFV infection were observed in the peripheral lymph nodes, gastro-duodenal junction, lung, or reproductive tract of any TAFV stock 1 NHPs (S5 Fig). We did not observe any TAFV immunoreactivity in samples from this group (S5 Fig). Similarly, no significant histopathologic lesions were observed in sections of nasal mucosa, conjunctiva, adrenal gland, or colon in either stock 1 or stock 2 NHPs.

**Fig 6:**
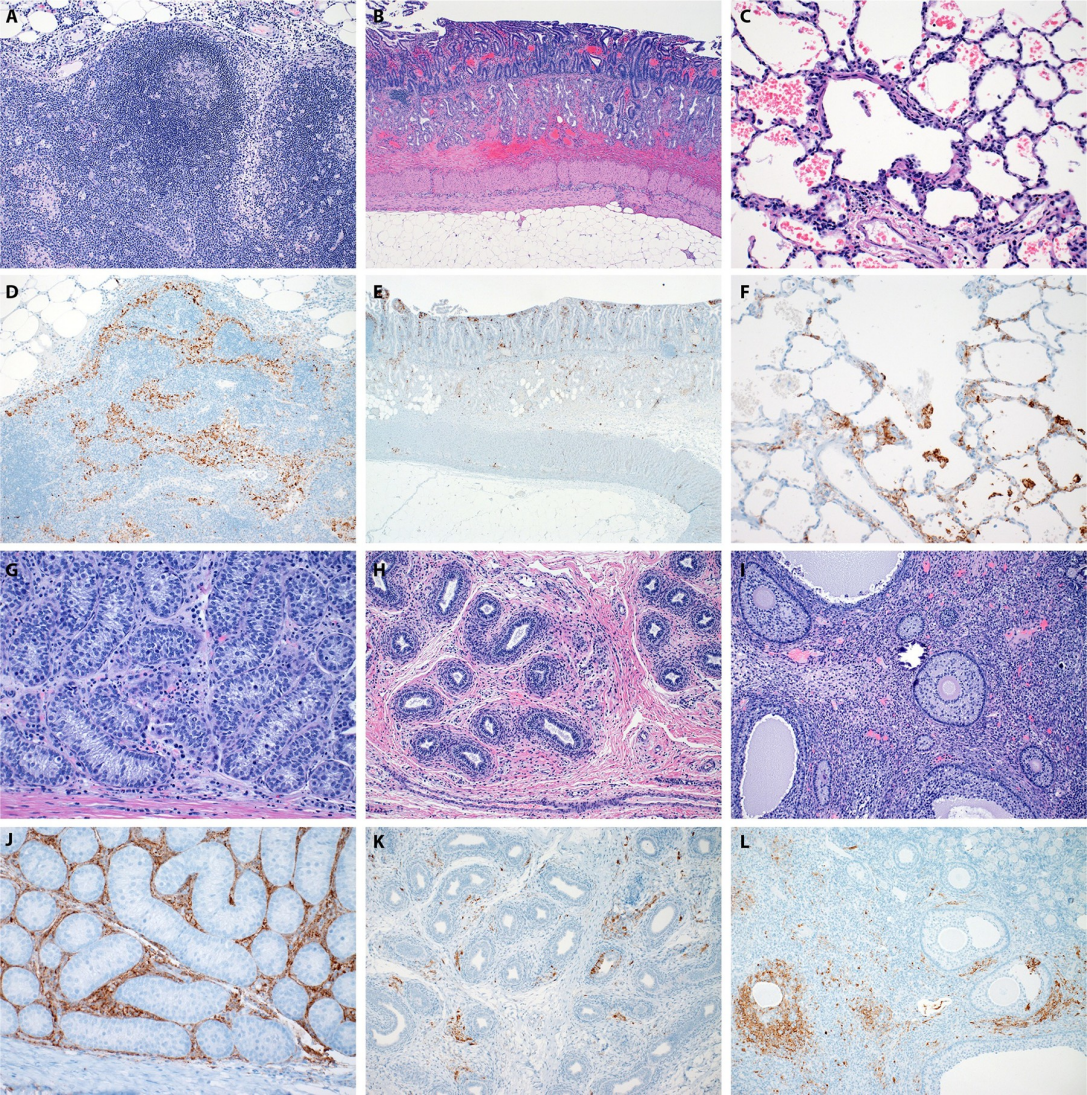
Histopathology of tissues from NHPs infected with TAFV stock 2. Tissue samples were collected at the time of euthanasia (8–11 dpi) and stained with hematoxylin & eosin (H&E) or for TAFV antigen immunoreactivity. (A,D) inguinal lymph node; (B,E) gastro-duodenal junction; (C,F) lung; (G,J) testicle; (H,K) epididymis; (I,L) ovary. All images are 100x.

In contrast, peripheral lymphoid tissues (axillary, inguinal, mediastinal, and mesenteric lymph nodes) in TAFV stock 2 NHPs exhibited lymphoid necrosis, sinus histiocytosis and rarely, erythrophagocytosis (not shown) in all NHPs (Fig 6A). A classic filovirus-associated lesion, gastro-duodenal mucosal hemorrhage, was noted in 4/4 of the stock 2 NHPs and was associated with superficial lymphohistiocytic duodenitis in 3/4 of these NHPs (both shown in Fig 6B). Minimal to mild interstitial pneumonia characterized by histiocytic and fewer lymphocytic interstitial infiltrates were noted in 3/4 of NHPs (Fig 6C). In addition, 1/4 of these NHPs had moderate pulmonary hemorrhage with alveolar fibrin rafts and perivascular fibrin accumulation. Evaluation of these systemic tissues by IHC showed TAFV antigen present in the subcapsular and medullary sinus histiocytes in the peripheral lymph nodes (Fig 6D), and infiltrating leukocytes within the lamina propria and submucosa and within mesenteric adipocytes surrounding the alimentary tract (Fig 6E). Within sections of the lung there was TAFV antigen present in endothelial cells, smooth muscle cells and/or pericytes, pulmonary and circulating macrophages and exceedingly rare pneumocytes associated with foci of interstitial pneumonia (Fig 6F).

There is growing evidence for potential sexual transmission of EBOV [9–11]; therefore, we assessed reproductive organs within the TAFV-infected NHPs. Overall, the reproductive tract lesions were ubiquitous in NHPs infected with TAFV stock 2. It is important to note that all of the males in the study were prepubescent. Histopathologic lesions were observed in only 1/3 of male gonads. This was limited to mild, interstitial, histiocytic and necrotizing orchitis (Fig 6G). Importantly, no histologic changes were observed within the seminiferous tubules of male NHPs. However, evaluated sections of the epididymis revealed a mild, perivascular and interstitial histiocytic epididymitis with minimal vacuolar degeneration of epithelial cells within the head and body of the epididymis of 3/3 stock 2 NHPs (Fig 6H). As none of the males were reproductively mature, spermatid maturation and abnormalities could not be assessed within the epididymis. The male gonads exhibited diffuse immunoreactivity in Leydig cells while no cells within seminiferous tubules exhibited immunoreactivity (Fig 6J). Within the epididymis, endothelial cells, pericytes, smooth muscle cells and infiltrating histiocytic cells expressed immunoreactivity in 3/3 males. In a single male, a focus of epithelial cells within the epididymal body exhibited distinct cytoplasmic immunoreactivity (Fig 6K). Mild histiocytic oophoritis was observed in the only female NHP infected with TAFV stock 2 (Fig 6I). Additionally, perivascular histiocytic cuffing and histiocytic infiltration limited to the myometrium were observed. Within the gonad of this female NHP, infiltrating histiocytic cells, luteal cells, thecal cells, and stromal cells all exhibited immunoreactivity (Fig 6L). Ocular pathologic changes were observed in 1/4 of stock 2 NHPs and were characterized by peracute choroidal separation with mild to moderate choroidal hemorrhage (S6 Fig). Inflammatory infiltrates were not observed within the eye of any evaluated NHP. Interestingly, immunoreactivity was observed in the choiroid, ciliary body and iris of 4/4 stock 2 inoculated NHPs (S6B and S6D Fig) and focally within the retina of 1/4 NHPs. These results indicate viral spread into the peripheral tissues and not just the primary filovirus target organs in NHPs that developed fatal disease. TAFV infection did not initiate a uniform cellular inflammatory influx in all tissue sets.

## Discussion

To date, there has only been one single human case of TAFV infection and disease described [2]. However, there are two TAFV NCBI reference sequences (GenBank NC_014372.1 and KU182910.1) originating from this patient in 1994. Our study compared two viral stock preparations with different genetic make-ups and the results indicate that the genome variations in these TAFV stocks result in different disease phenotypes. The different TAFV genome sequences are likely the result of the virus acquiring mutations during passaging in cell culture in the presence or absence of mycoplasma treatment. We found that the sequence of TAFV stock 1 was similar to the NCBI GenBank reference sequence NC_014372.1 while TAFV stock 2 shared a greater homology with reference sequence KU182910.1. The sequence differences were detected throughout the noncoding and coding regions of the genome including the GP, VP30, and the L. Differences between the TAFV genomes in VP30 and L; both being components of the ribonucleoprotein complex (RNP), may be impacting the viral replication efficiency. For EBOV VP30, it has been shown that it can regulate viral replication depending on its phosphorylation state which determines its transcription activation function [12]. Phosphorylation occurs within the N-terminal 60 amino acids of serine and threonine residues and one of the current mutations in the TAFV VP30 is located in this area, possibly contributing to differences in viral replication. Unfortunately, there are no reverse genetics systems; such as the minigenome system, available for TAFV to generate recombinant viruses and investigate the impact of each one of these mutations on viral pathogenicity. Future efforts will hopefully close this gap and enable studies addressing the impact of these mutations *in vitro* and *in vivo*.

Ebolavirus GP genes, including TAFV GP, encode 3 proteins: GP_1,2_, soluble GP (sGP), and small soluble GP (ssGP). sGP is the primary translation product of GP expressed from unedited RNA transcripts while GP_1,2_ and ssGP are products of a unique co-transcriptional editing process. Transcripts that encode GP_1,2_ contain 8 adenosine residues (8A) while sGP transcripts contain 7A and ssGP transcripts contain either 6A or 9A [13]. It has been shown that through passaging on Vero E6 cells the EBOV genotype changed from 7U to 8U only after 5 passages, indicating that there is selective pressure in cell culture [14]. The exact passage number of the TAFV stock 1 before we received it is unknown to us, however, we do know that the working stock that we used is greater than 5 passages while TAFV stock 2 is passage 2. The TAFV stock 2 that caused severe disease in NHPs had a guanine in this uracil editing site; however, all NHPs who succumbed to infection with this virus had 7U which indicated that the virus reverted and potentially impacted viral editing. Future studies are needed to confirm this hypothesis.

The IFNAR^-/-^ mouse study demonstrated that i.p. inoculation of TAFV led to infection, however, it did not cause disease. This is similar to an early study describing 100% survival for TAFV infection in this mouse model with 100 PFU i.p. or s.c. [15]. The current TAFV stocks were wildtype and not rodent-adapted, which likely contributed to the limited viral replication and lack of disease in this mouse model. Both of the mouse-adapted Makona and Mayinga variants of EBOV contained a VP24 mutation important for virulence that the current wildtype TAFV isolates lacked [16]. Future studies could create a mouse-adapted TAFV stock if further studies are to be performed in IFNAR^-/-^ mice. However, TAFV exposure in the IFNAR^-/-^ model is not the first instance where wildtype filoviruses did not cause disease as shown by a previous study with Lloviu virus and Bombali virus [17] and earlier studies demonstrating inconsistent disease after filovirus infection even between isolates from the same virus species [15]. A study performed in humanized (huNSG-A2 mice) mice demonstrated 81.8% survival after TAFV infection with only low levels of viremia [18]. A more recent study in ferrets showed only mild signs of disease after infection and 100% survival [19]. Interestingly, this ferret study used the parent of our TAFV stock 1 that we propagated for our use. As for TAFV infection in the cynomologus macaque model, the findings herein revealed classical filovirus disease only with TAFV stock 2. Even though both stocks originated from the single human case, genomic differences between the two stocks impacted TAFV virulence. However, the variability in disease severity and lethality achieved by TAFV infection in the different animal models highlights the importance of identifying the correct animal model for assessing differences in viral pathogenicity and the purpose of scientific studies.

The transcriptional analyses of samples collected from both NHP groups supported the clinical findings of our study, revealing an inflammatory response only for NHPs infected with TAFV stock 2. There was a clear distinction between the NHPs in both groups as shown by the MaSigPro analysis. The clusters demonstrate an escalating inflammatory response in the NHPs that developed lethal disease and a recovery from limited infection in the surviving NHPs. A large number of upregulated DEGs in the TAFV stock 1 NHPs mapped to GO terms associated with processes indicative of viral infection resolution, such as cell cycle, cell killing, and DNA damage and repair. These findings are in line with observations made in ebola virus disease survivors which have NK cell accumulation compared to non-survivors [20]. These longitudinal transcriptional underpinnings mirrored the inflammatory markers measured within the serum. Some of the stock 1 NHPs had slight increases of IL-1ra, MCP-1, and IL-1β between 6 and 8 dpi which recovered by 11 dpi as also indicated in the transcriptional analyses. In contrast, stock 2 NHPs demonstrated with an increase of all inflammatory markers overtime without recovery as highlighted as a “cytokine storm”, a hallmark of classical filovirus disease [21]. Together, this data suggests a more controlled response in animals inoculated with TAFV stock 1 and a response similar to hemorrhagic fever virus in animals inoculated with TAFV stock 2 [22,23]. Furthermore, fatal EBOV infection is associated with significant transcriptional changes in myeloid cells which are consistent with changes that were only observed in TAFV infection due to the stock 2 virus [24,25].

In addition to gaining insight into the different disease phenotypes achieved after inoculation with both TAFV stocks, we were able to analyze histopathologic samples in depth. Interestingly, IHC revealed an abundance of viral antigen in tissues and organs in which no significant histopathologic lesions were observed. This was particularly striking in the testicle of all 3 male NHPs infected with TAFV stock 2 in which nearly ubiquitous immunoreactivity was observed in Leydig cells and only a mild, focal orchitis was noted in one male. Similarly, immunoreactivity was readily in the choroid, ciliary body, and iris of all stock 2 NHPs without any evidence of histologic evidence of inflammation and also noted in adipocytes, particularly those within peri-duodenal mesentery, without any indication of inflammatory influx. Viral spread and dissemination to these tissues is poorly understood and may be an area of future research. A limitation of the current study is an unequal sex distribution, so further analysis should examine potential sex differences. This is of particular interest since there is a report of a EBOV disease survivor who sexually transmitted EBOV in 2016 [9] and a previous study in rhesus macaques showed EBOV infection in reproductive organs [10]. Additionally, immunoreactive cells showed no morphologic changes associated with degeneration, apoptosis, necroptosis, or another cell death pathway. Due to the NHPs meeting euthanasia criteria, it is unknown if these sites would eventually undergo inflammatory infiltration with activation of cell death pathways, or if there is a mechanism for viral persistence and immune evasion in these tissues.

Interstitial pneumonia is an infrequently reported histologic change associated with experimental infection of filoviruses in the NHP model. Minimal to mild interstitial pneumonia associated with abundant and widespread viral immunoreactivity was noted in all NHPs inoculated with TAFV stock 2. Additionally, one NHP had grossly observed hemorrhage that was confirmed as an ante-mortem change by histopathologic evaluation. Interestingly, interstitial pneumonia is an uncommon clinical disease state associated with filovirus disease and may be underreported in humans due to the severity of other systemic changes. However, clinical interstitial pneumonia has been recognized and documented in one human case during the West African EBOV epidemic [26]. While the histologic changes are minimal to mild, subclinical interstitial pneumonia may be a contributory factor to the severity of filovirus clinical disease.

In conclusion, the studies described herein confirm the limited value of IFNAR^-/-^ mice as a disease model for TAFV infection and potentially for other filovirus infections as well. In contrast, we add to the limited number of studies describing TAFV pathogenesis in cynomolgus macaques. Finally, we highlight that viral passage history and genotypic differences for filoviruses can greatly impact disease development in an animal model. Future work will include establishment and investigation of recombinant TAFV *in vitro* and *in vivo* to decipher molecular underpinnings of pathogenesis.

## Materials and methods

### Ethics statement

All infectious work with filoviruses was performed following standard operating procedures (SOPs) approved by the Rocky Mountain Laboratories (RML) Institutional Biosafety Committee (IBC) in the maximum containment laboratory at RML, Division of Intramural Research, National Institute of Allergy and Infectious Diseases, National Institutes of Health. Animal work was performed in accordance with the recommendations described in the Guide for the Care and Use of Laboratory Animals of the National Institute of Health, the Office of Animal Welfare and the United States Department of Agriculture and was approved by the RML Animal Care and Use Committee (ACUC). Procedures were conducted in animals anesthetized by trained personnel under the supervision of veterinary staff. Food and water were available *ad libitum*. Macaques were housed in adjoining individual primate cages that enabled social interactions, under controlled conditions of humidity, temperature, and light (12h light:12h dark cycles). They were monitored and fed commercial monkey chow, treats, and fruit at least twice a day by trained personnel. Environmental enrichment consisted of commercial toys, music, and video. For all animal studies, endpoint criteria were specified by RML ACUC-approved parameters to determine when animals were humanely euthanized.

### Cell lines and viruses

VeroE6 cells were grown at 37°C and 5% CO_2_ in Dulbecco’s modified Eagle’s medium (DMEM) (Sigma-Aldrich, St. Louis, MO) containing 10% fetal bovine serum (FBS) (Wisent Inc., St. Bruno, Canada), 2 mM L-glutamine, 50 U/mL penicillin, and 50 μg/mL streptomycin (all Thermo Fisher Scientific, Waltham, MA). Both TAFV stocks were isolated from a sample originating from a human patient from the Republic of Côte d’Ivoire in 1994 [27]. TAFV stock 1 (>5 passages) was received from the Public Health Agency of Canada [19]. The virus was contaminated with mycoplasma and cleaned up in our laboratory using Mycoplasma-EX Kit (PromoKine, Heidelberg, Germany) according to manufacturer’s specifications. TAFV stock 2 (passage 2) was obtained from Dr. Thomas Ksiazek, University of Texas Medical Branch, was mycoplasma negative, and used directly for infection of macaques [8]. Reston virus (RESTV) and EBOV were mycoplasma negative and used as previously described [28]. Deep sequencing of all virus stocks revealed no contaminants; however, multiple base pair changes were noted when the obtained sequences for the TAFV stocks were compared to existing reference sequences NC_014372 and KU182910 (S1 Table). The sequence of TAFV stock 1 has higher homology to reference sequence NC_014372 while the TAFV stock 2 sequence has higher homology to reference sequence KU182910.

### TAFV growth kinetics

A confluent monolayer of Vero E6 cells was infected (multiplicity of infection 0.1) in triplicate with either TAFV stock 1 or stock 2 in plain DMEM in a 24-well plate and incubated 1 hour at 37°C. Then, viral inoculums were removed and the cells were washed 3 times with plain DMEM (0.5 mL/wash). Finally, fresh DMEM supplememnted with 2% FBS was added (0.5 mL/well). Cells were incubated at 37°C and monitored for cytopathic effect up to 7 dpi. A sample was collected from each well every 24 hours and titered on Vero E6 cells. Titers were calculated as median tissue culture infectious doses (TCID_50_)/mL using the Reed-Muench method.

### IFNAR^-/-^ mouse studies

On 0 dpi, male & female adult (8–13 weeks old) C57BL/6 IFNAR^-/-^ mice (B6.129S2-Ifnar1tm1Agt/Mmjax; n = 14/group) were infected (200 μL/mouse) via the intraperitoneal (i.p.) route with 100 TCID_50_ (low dose) of TAFV stock 1, stock 2, or EBOV or with 100,000 TCID_50_ (high dose) of TAFV stock 1, stock 2, or RESTV. At a late stage of disease for EBOV (6 dpi), 4 mice per group were euthanized for sample collection. A survival cohort per group (n = 6) was euthanized at the study end (28 dpi). RESTV infection served as “no disease control” and EBOV infection as “disease control” [17]. The mice were observed at least twice daily for clinical signs of disease and humanely euthanized when they reached the RML ACUC-approved endpoint criteria. Mice were weighed once/day until 10 dpi and surviving mice were euthanized at the study end point (28 dpi).

### NHP study

Eight cynomolgus macaques (*Macaca fascicularis*; 5 male and 3 female) of Cambodian origin, 3–4 years old and 2.3–5.0 kg in weight were used for this study. On 0 dpi, macaques were challenged i.m. with 10,000 TCID_50_ (equaling 1,000 PFU) of either TAFV stock 1 (n = 4) or TAFV stock 2 (n = 4) [6]. Physical examinations and blood draws were performed on 0, 1, 3, 6, 8, 11, 14, 21, 28 dpi. The macaques were observed at least twice daily for clinical signs of disease according to an RML ACUC-approved scoring sheet and humanely euthanized when they reached endpoint criteria. The study end point was 28 dpi when all surviving macaques were humanely euthanized. Parts of the data of TAFV stock 2 macaques has previously been published [8].

### RNA extraction and RT-qPCR

RNA from whole blood (EDTA) and swabs (oral, nasal, rectal, and urogenital collected in DMEM without supplements) samples were extracted with the QIAmp Viral RNA Mini Kit (Qiagen, Hilden, Germany). RNA from tissue samples (maximum of 30 mg/tissue) was extracted with the RNeasy Mini Kit (Qiagen) according to manufacturer’s specifications. One-step RT-qPCR was performed with the QuantiFast Probe RT-PCR+ROX Vial Kit (Qiagen) with a specific TAFV-L primer-probe set as described previously [19] on the Rotor-Gene Q (Qiagen). RNA from the TAFV stocks was extracted via QIAmp Viral RNA Mini Kit (Qiagen) and used alongside samples as standards with known TCID_50_ concentrations (TCID_50_ equivalents).

### Enzyme-linked immunosorbent assay (ELISA)

Serum was inactivated by γ-irradiation (4 MRad) [29] and removed from the maximum containment laboratory according to SOPs approved by the IBC. For mice: ELISA was performed as previously described [30] using purified TAFV glycoprotein (GP) as an antigen. For NHPs: TAFV GP-specific IgG levels were measured in serum samples at a 1:1,000 dilution using the human anti-TAFV GP IgG ELISA kit (Alpha Diagnostic International, San Antonio, TX) according to manufacturer’s specifications. The kit was modified by using the anti-monkey IgG HRP secondary antibody (Alpha Diagnostic International) instead of anti-human IgG HRP. IFNα levels were measured in undiluted serum samples using the VeriKine Cynomolgus/Rhesus IFN Alpha Serum ELISA kit (PBL, Piscataway, NJ) according to manufacturer specifications.

### Hematology and serum biochemistry

Blood samples were collected in tubes containing EDTA and the total cell counts were determined via Idexx ProCyte DX analyzer (Idexx Laboratories, Westbrook, ME). Serum biochemistry was analyzed on a Vetscan 2 using Preventive care profile disks (Abaxis, Union City, CA).

### Virus neutralization assay

The neutralization capacity of γ-irradiated and heat-inactivated serum samples was assessed in VeroE6 cells using VSV-TAFV-GFP as previously described [31]. The GFP-positive cell count was determined on the FACSymphony A5 Cell Analyzer (BD Biosciences, Mississauga, ON, Canada). Data were analyzed using FlowJo V10.

### Serum cytokine and chemokine levels

Serum samples were assessed using the Milliplex MAP NHP cytokine magnetic bead kit (Millipore, Burlington, MA) by diluting in serum matrix (1:2) according to manufacturer specifications. Samples were read on the Bio-Plex 200 system (Bio-Rad, Hercules, CA).

### Histopathology and immunohistochemistry (IHC)

Necropsies and tissue sampling were performed according to IBC-approved SOPs. Harvested tissues were processed as previously described [8]. Immunoreactivity was detected using a previously described cross-reactive anti-EBOV VP40 antibody at a 1:1000 dilution [32,33]. All tissue slides were evaluated by a board-certified veterinary pathologist.

### Library preparation and sequencing

Libraries were generated via the NEBNext Ultra II Directional RNA Library Prep Kit per manufacturer instructions with rRNA depletion by the NEBNext rRNA Depletion kit (New England Biolabs, Ipswich, NE). An Agilent 2100 Bioanalyzer was used to assess cDNA library quality and concentration before multiplexing and sequencing on the Illumina NextSeq 2000 platfrorm.

### Bioinformatics

The Trim Galore package was used to trim raw sequences to 70 bp and an average Phred score of 30. Trimmed sequences were aligned to the *Macaca fascicularis* genome “Macaca_fascicularis.Macaca_fascicularis_6.0.dna.toplevel.fa” using hisat. Genes were annotated with Ensembl file “Macaca_fascicularis.Macaca_fascicularis_6.0.106.gtf”. Raw gene counts were identified by the summarizeOverlaps package before being converted to transcripts per kilobase million (TPM).

Gene expression analysis was performed using three approaches: (1) EdgeR, (2) STEM, and (3) MaSigPro [34–36]. The EdgeR package identifies differentially expressed genes (DEGs) in one condition relative to another. All DEGs were identified relative to 0 dpi. DEGs were filtered for only those with an FDR ≤ 0.05, log2 fold change ≤ −1 or ≥ 1, and encoded for human protein-coding homologs. The Short Time Series Expression Miner (STEM) software was used to identify significant patterns of longitudinal gene expression change [34] within each condition. The MaSigPro package was used to identify significant longitudinal gene expression profile changes between conditions using a two-way forward regression strategy. The 11 dpi data set from TAFV stock 2 includes all 4 macaques at time of euthanasia. Gene ontology (GO) terms were identified using Metascape [37]. All heatmaps, bar plots, bubble plots, and Venn diagrams were made using R package ggplot2.

### Statistical analysis

Log-rank (Mantel-Cox) test was performed for survival rates between animal groups. Two-way ANOVA with Tukey’s multiple comparisons test was performed between IFNAR^-/-^ mouse viral load groups. All analyses were performed using GraphPad Prism Software (v. 9.3.1).

## Supporting information

S1 Table TAFV sequence differences. (PDF)

S1 Fig Growth kinetics of TAFV stocks in Vero E6 cells. (PDF)

S2 Fig Serum cytokine and chemokine levels in NHPs after TAFV infection. (PDF)

S3 Fig Transcriptional response in TAFV stock 1 NHPs. (PDF)

S4 Fig STEM analysis of transcriptional changes in stock 2 NHPs. (PDF)

S5 Fig Histopathology and IHC in NHP tissues infected with TAFV stock 1. (PDF)

S6 Fig Ocular pathologic changes in TAFV stock 2-infected NHP. (PDF)
