# Supplementary material for: Pathogenic differences of cynomolgus macaques after Taï Forest virus infection depend on the viral stock propagation
Source: PLoS Pathog. 2024 Jun 11;20(6):e1012290. doi: 10.1371/journal.ppat.1012290 (PMC11195944; doi:10.1371/journal.ppat.1012290)
Supplement: S1 Table — (PDF) [file ppat.1012290.s001.pdf]

**S1 Table. TAFV sequence differences.**

| Gene | Location (bp) | Stock | Nucleic Acid | Coding Region | Amino Acid |
|---|---|---|---|---|---|
| **NP** | 2719 | TAFV stock 2 | T | No | n/a |
| **VP40** | 5771 | NC_014372 | C | No | n/a |
| | | TAFV stock 1 | C | No | n/a |
| | | KU182910 | T | No | n/a |
| | | TAFV stock 2 | T | No | n/a |
| | 5789 | NC_014372 | C | No | n/a |
| | | TAFV stock 1 | C | No | n/a |
| | | KU182910 | T | No | n/a |
| | | TAFV stock 2 | T | No | n/a |
| **GP** | 6322 | NC_014372 | G | Yes | C |
| | | TAFV stock 1 | G | Yes | C |
| | | KU182910 | A | Yes | Y |
| | | TAFV stock 2 | A | Yes | Y |
| | 6427 | NC_014372 | G | Yes | R |
| | | TAFV stock 1 | G | Yes | R |
| | | KU182910 | A | Yes | H |
| | | TAFV stock 2 | A | Yes | H |
| | 6907 | TAFV stock 2 | G | Yes | E |
| | 6918 | KU182910 | A | Yes | K |
| **VP30** | 8600 | NC_014372 | C | Yes | A |
| | | TAFV stock 1 | C | Yes | A |
| | | KU182910 | A | Yes | D |
| | | TAFV stock 2 | A | Yes | D |
| | 9762 | NC_014372 | A | No | n/a |
| **L** | 12256 | NC_014372 | A | Yes | I |
| | | TAFV stock 1 | A | Yes | I |
| | | KU182910 | G | Yes | V |
| | | TAFV stock 2 | G | Yes | V |
| | 16625 | NC_014372 | C | Yes | T |
| | | TAFV stock 1 | C | Yes | T |
| | | KU182910 | A | Yes | N |
| | | TAFV stock 2 | A | Yes | N |
