# Supplementary material for: Pathogenic differences of cynomolgus macaques after Taï Forest virus infection depend on the viral stock propagation
Source: PLoS Pathog. 2024 Jun 11;20(6):e1012290. doi: 10.1371/journal.ppat.1012290 (PMC11195944; doi:10.1371/journal.ppat.1012290)
Supplement: S1 Fig — (PDF) [file ppat.1012290.s002.pdf]

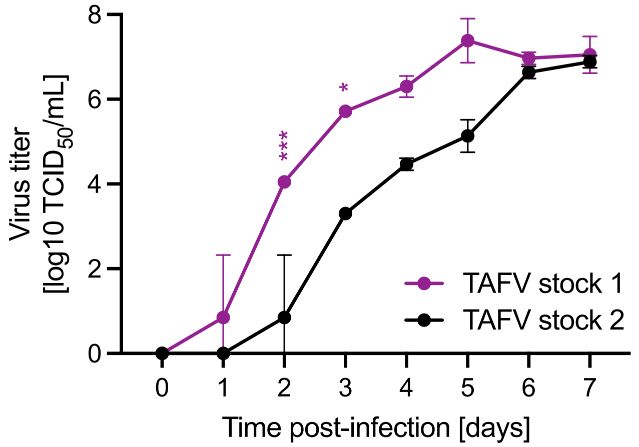

S1 Fig. Growth kinetics of TAFV stocks in Vero E6 cells. Cells were infected with either TAFV stock 1 or stock 2 at a multiplicity of infection of 0.1 in triplicate and samples were collected every 24 hours. Titrations were performed on Vero E6 cells to determine viral titers at each timepoint. Data are displayed as geometric mean and geometric standard deviation. Statistical significance determined by two-way ANOVA is indicated as *p>0.05 and ***p>0.001.
