# Supplementary material for: Pathogenic differences of cynomolgus macaques after Taï Forest virus infection depend on the viral stock propagation
Source: PLoS Pathog. 2024 Jun 11;20(6):e1012290. doi: 10.1371/journal.ppat.1012290 (PMC11195944; doi:10.1371/journal.ppat.1012290)
Supplement: S2 Fig — (PDF) [file ppat.1012290.s003.pdf]

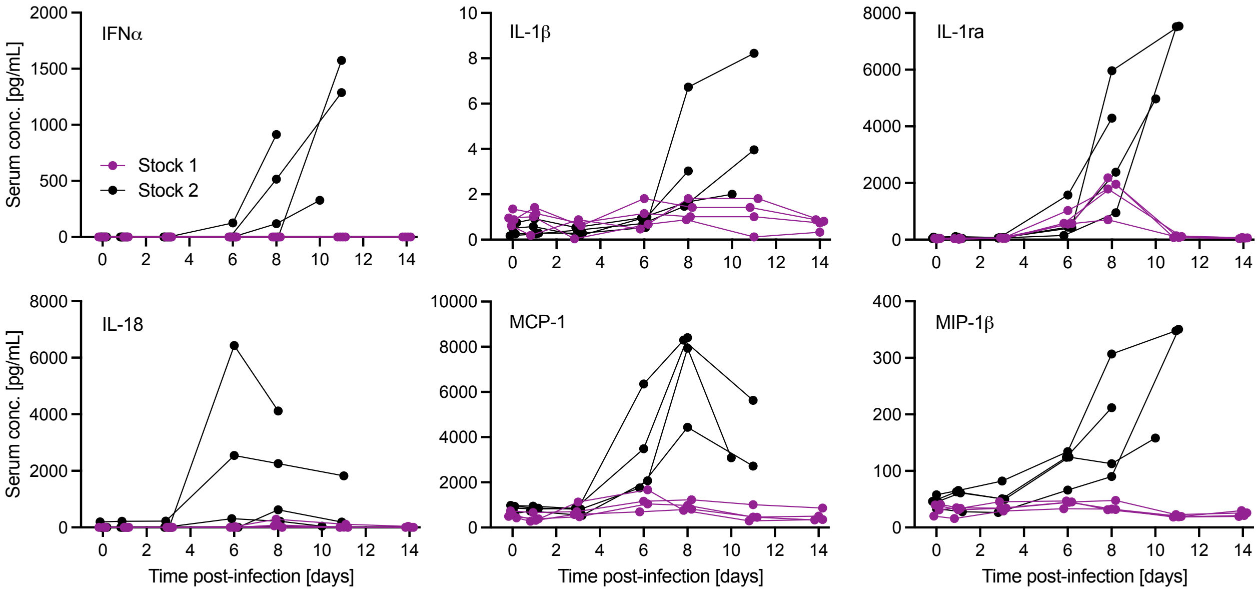

S2 Fig. Serum cytokine and chemokine levels in NHPs after TAFV infection. Levels of select cytokines and chemokines in serum samples collected from each NHP.
