# Supplementary material for: Pathogenic differences of cynomolgus macaques after Taï Forest virus infection depend on the viral stock propagation
Source: PLoS Pathog. 2024 Jun 11;20(6):e1012290. doi: 10.1371/journal.ppat.1012290 (PMC11195944; doi:10.1371/journal.ppat.1012290)
Supplement: S3 Fig — (PDF) [file ppat.1012290.s004.pdf]

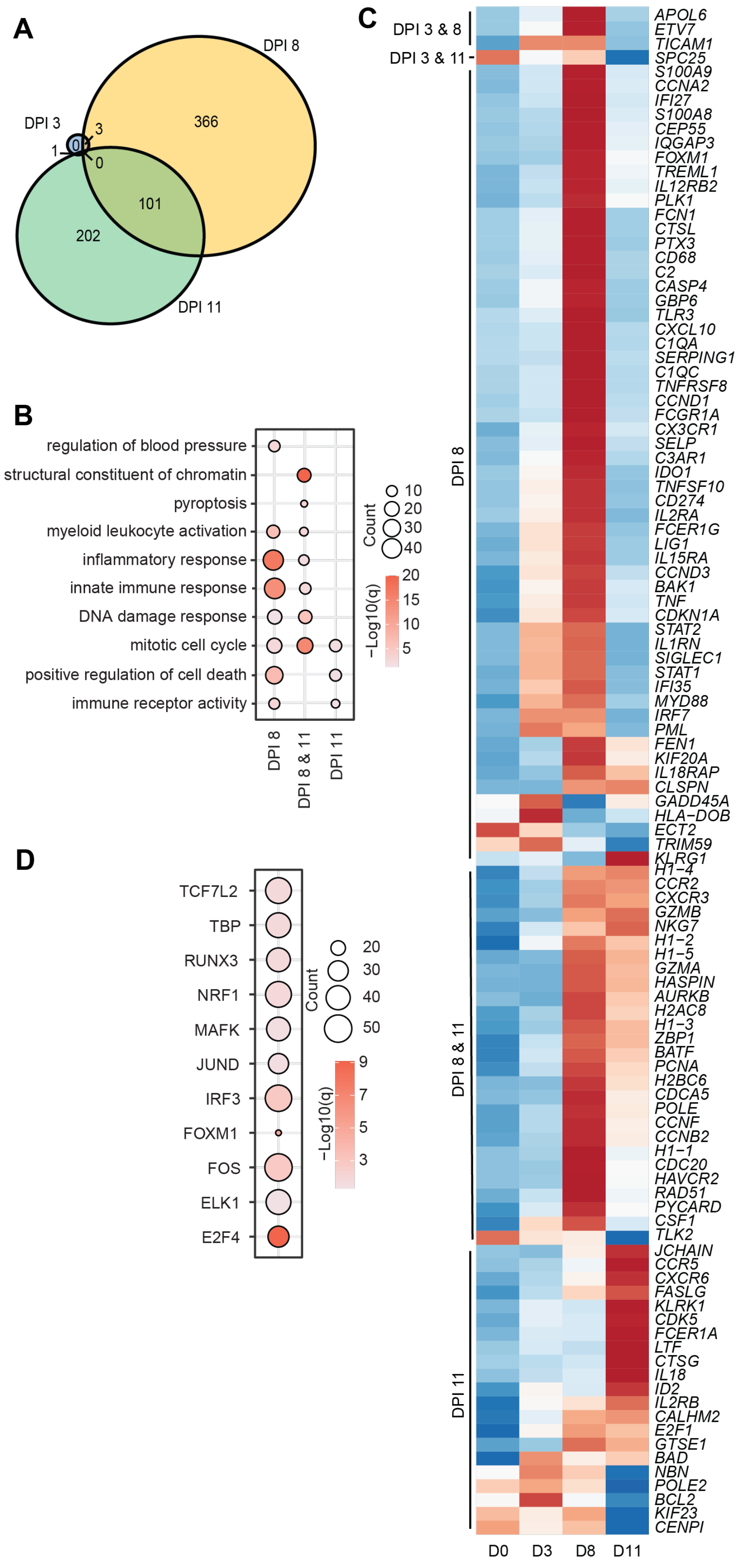

S3 Fig. Transcriptional response in TAFV stock 1 NHPs. (A) Venn diagram of DEG identified at 3, 8, and 11 dpi relative to 0 dpi. (B) Bubbleplot representing GO terms for DEGs in panel B. Color indicates –log10(q) and size indicates the number of genes within the GO term. (C) Heatmap of average TPM values. The dpi is indicated on the left. (D) Bubbleplot representing transcription factors DEGs unique to 11 dpi. Color indicates – log10(q) and size indicates the number of genes.
