# Supplementary material for: Pathogenic differences of cynomolgus macaques after Taï Forest virus infection depend on the viral stock propagation
Source: PLoS Pathog. 2024 Jun 11;20(6):e1012290. doi: 10.1371/journal.ppat.1012290 (PMC11195944; doi:10.1371/journal.ppat.1012290)
Supplement: S4 Fig — (PDF) [file ppat.1012290.s005.pdf]

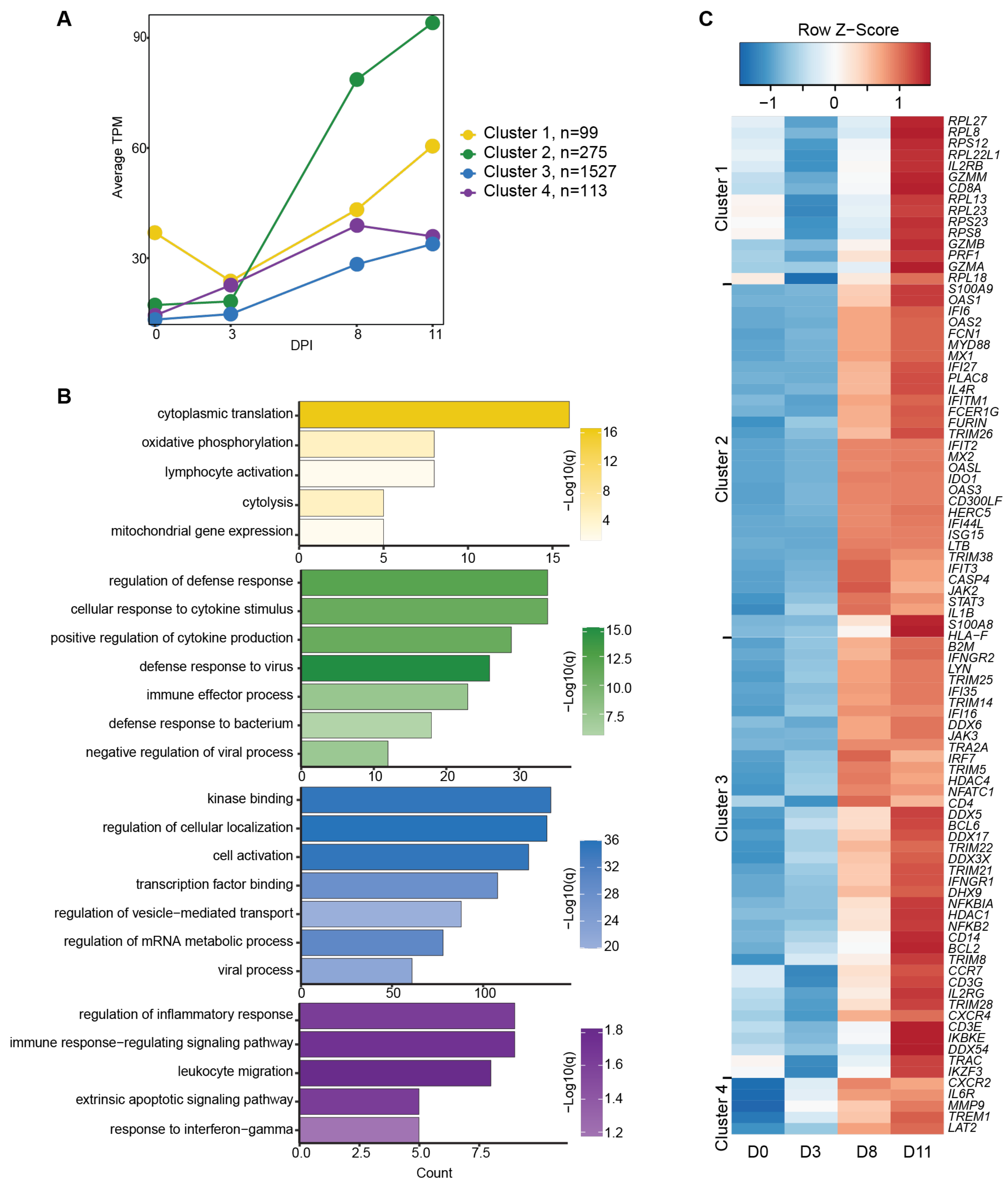

S4 Fig. STEM analysis of transcriptional changes in stock 2 NHPs. (A) Average TPM of the four gene expression clusters identified by STEM over time. (B) Bar plot representing GO terms for genes from cluster 1 (yellow), cluster 2 (green), cluster 3 (blue), and cluster 4 (purple) in panel A. Color indicates -log10(q) and length indicates the number of genes within the GO term. (C) Heatmap of average TPM values for selected genes mapping to GO terms in Panel B. Color is based on scaled and centered TPM values.
