# Supplementary material for: Pathogenic differences of cynomolgus macaques after Taï Forest virus infection depend on the viral stock propagation
Source: PLoS Pathog. 2024 Jun 11;20(6):e1012290. doi: 10.1371/journal.ppat.1012290 (PMC11195944; doi:10.1371/journal.ppat.1012290)
Supplement: S5 Fig — (PDF) [file ppat.1012290.s006.pdf]

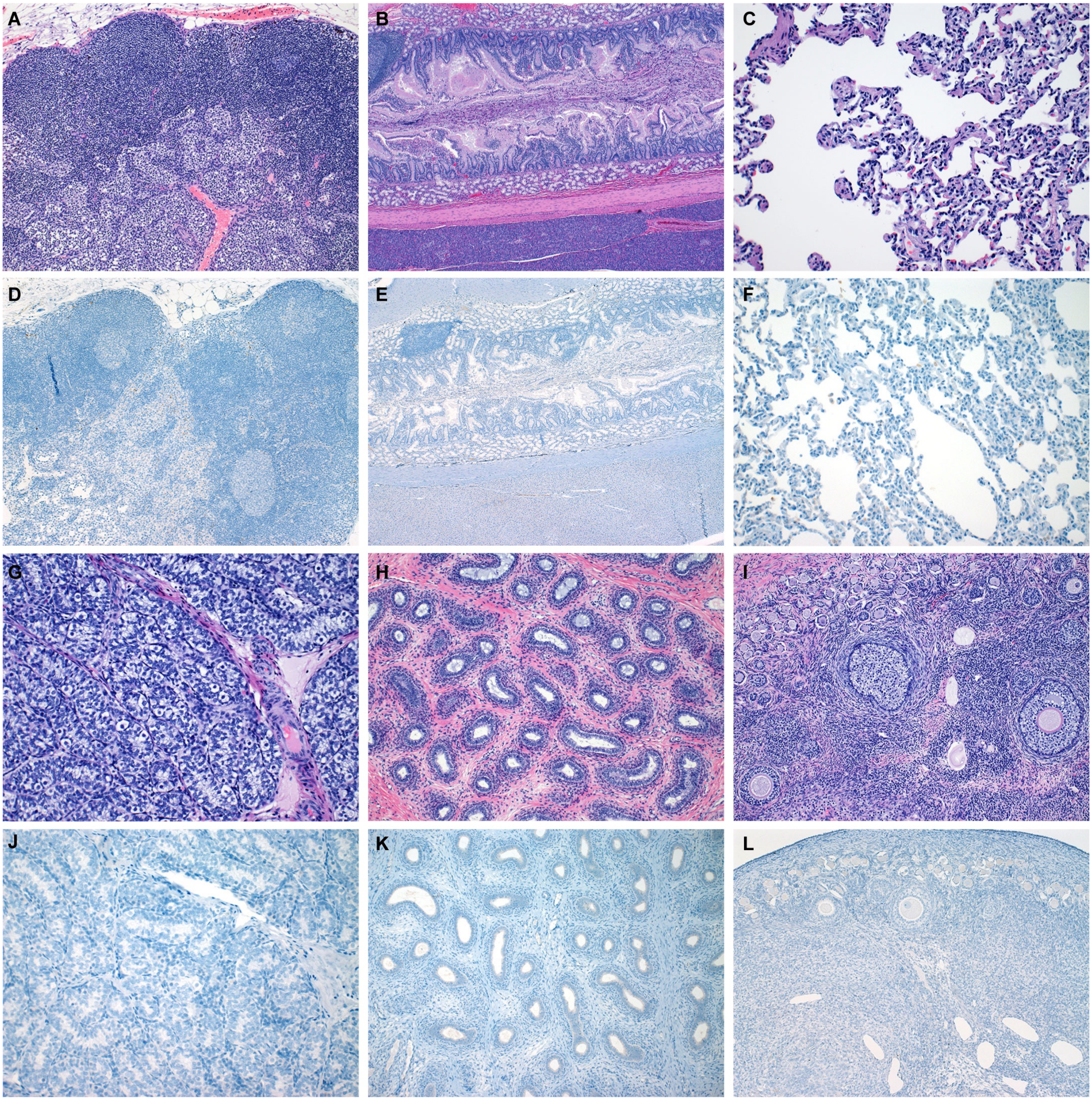

S5 Fig. Histopathology and IHC in NHP tissues infected with TAFV stock 1. Tissue samples were collected at the time of euthanasia (28 dpi) and stained with H&E or for TAFV antigen immunoreactivity. (A,D) inguinal lymph node; (B,E) gastro-duodenal junction; (C,F) lung; (G,J) testicle; (H,K) epididymis; (I,L) ovary. All pictures are 100x.
