# Supplementary material for: Pathogenic differences of cynomolgus macaques after Taï Forest virus infection depend on the viral stock propagation
Source: PLoS Pathog. 2024 Jun 11;20(6):e1012290. doi: 10.1371/journal.ppat.1012290 (PMC11195944; doi:10.1371/journal.ppat.1012290)
Supplement: S6 Fig — (PDF) [file ppat.1012290.s007.pdf]

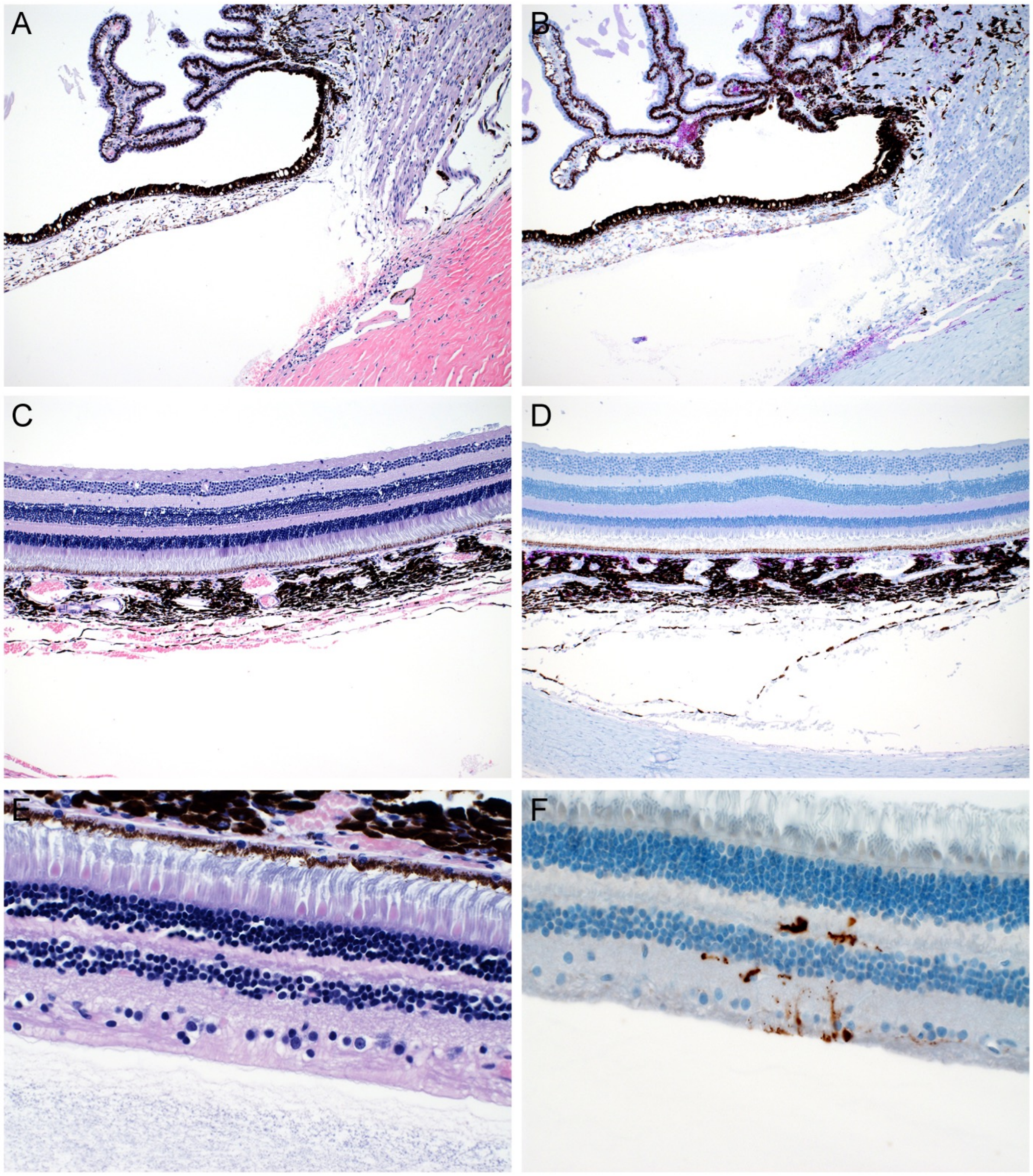

S6 Fig. Ocular pathologic changes in TAFV stock 2-infected NHPs. Tissue samples were collected at the time of euthanasia (8-11 dpi) and stained with H&E or TAFV antigen by IHC. (A) Iridocorneal angle with anterior chamber, posterior chamber, iris, ciliary body and a portion of the sclera (100x, H&E). (B) Antigen primarily within the iridocorneal angle and the ciliary body (100x, IHC). (C) Expansion of the choroid by hemorrhage separating the choroid from the sclera (100x, H&E). (D) Antigen primarily within the choird, choriocapillary layer and rarely within the pigmented epithelium (100x, IHC). (E) Retina from a stock 2-infected NHP (400x, H&E). (F) Immunoreactivity noted in the nerve fiber, inner plexiform and outer plexiform laters of the retina (400x, IHC).
